# Sex-biased gene expression at single-cell resolution: cause and consequence of sexual dimorphism

**DOI:** 10.1093/evlett/qrad013

**Published:** 2023-04-14

**Authors:** Iulia Darolti, Judith E Mank

**Affiliations:** Department of Zoology and Biodiversity Research Centre, University of British Columbia, Vancouver, BC, Canada; Department of Ecology and Evolution, University of Lausanne, Lausanne, Switzerland; Department of Zoology and Biodiversity Research Centre, University of British Columbia, Vancouver, BC, Canada

**Keywords:** scRNA-sequencing, differential expression, tissue composition, allometry, regulatory evolution

## Abstract

Gene expression differences between males and females are thought to be key for the evolution of sexual dimorphism, and sex-biased genes are often used to study the molecular footprint of sex-specific selection. However, gene expression is often measured from complex aggregations of diverse cell types, making it difficult to distinguish between sex differences in expression that are due to regulatory rewiring within similar cell types and those that are simply a consequence of developmental differences in cell-type abundance. To determine the role of regulatory versus developmental differences underlying sex-biased gene expression, we use single-cell transcriptomic data from multiple somatic and reproductive tissues of male and female guppies, a species that exhibits extensive phenotypic sexual dimorphism. Our analysis of gene expression at single-cell resolution demonstrates that nonisometric scaling between the cell populations within each tissue and heterogeneity in cell-type abundance between the sexes can influence inferred patterns of sex-biased gene expression by increasing both the false-positive and false-negative rates. Moreover, we show that, at the bulk level, the subset of sex-biased genes that are the product of sex differences in cell-type abundance can significantly confound patterns of coding-sequence evolution. Taken together, our results offer a unique insight into the effects of allometry and cellular heterogeneity on perceived patterns of sex-biased gene expression and highlight the power of single-cell RNA-sequencing in distinguishing between sex-biased genes that are the result of regulatory change and those that stem from sex differences in cell-type abundance, and hence are a consequence rather than a cause of sexual dimorphism.

## Introduction

Males and females of the same species often exhibit striking differences in a broad range of phenotypic traits, despite sharing the majority of their genome. Changes in patterns of gene expression between the sexes are therefore thought to be key to the evolution of sexual dimorphism (Grath & Parsch, 2016; Mank, 2017), and sex-biased genes are often used as a way to measure the signature of sex-specific selection within the genome. Indeed, a large body of work indicates that the magnitude of transcriptional sex differences correlates with the degree of phenotypic dimorphism across multiple levels of biological diversity (Djordjevic et al., 2022; Ingleby et al., 2015; Magnusson et al., 2011; Mank et al., 2010; Perry et al., 2014; Pointer et al., 2013). However, distinguishing between the causal and the dependent variable has important implications for studying gene expression evolution and for inferences of sexual selection and sexual conflict.

Testing for the causal effect of sex-biased gene expression on phenotypic dimorphism is nontrivial (Abzhanov et al., 2006; Chen et al., 2015; Galouzis & Prud’homme, 2021; Khila et al., 2012; Toubiana et al., 2021). Functional genetic assays are limited by the sheer number of sex-biased loci, the polygenic nature of traits, or simply because the necessary genetic tools are lacking, a particular problem for non-model organisms, which often present the most striking phenotypic differences. Moreover, the assumption that sex-biased genes are the result of regulatory rewiring may not always hold true. Most differential expression analyses have so far relied on bulk RNA-sequencing, comparing expression level between organs (Harrison et al., 2015; Whittle & Extavour, 2019), whole body parts comprising various tissues such as heads or abdomens (Immonen et al., 2017; Standage et al., 2016), or even entire organisms (Djordjevic et al., 2022; Hollis et al., 2014; Ranz et al., 2003; Stuglik et al., 2014). This approach averages expression across a complex aggregate of diverse cell types, ignoring the stochasticity of gene expression across cell populations. In many species, males and females exhibit dimorphism in the relative size of their constituent body parts (Badyaev, 2002), and allometric scaling could influence perception of differential expression in studies where whole organisms are used for RNA preparation (Montgomery & Mank, 2016). In a similar way, whole tissue expression studies may be affected by the heterogeneity in cell-type abundance or composition (Fair et al., 2020; Fuess & Bolnick, 2021; Hunnicutt et al., 2022; Price et al., 2022), and there is indeed evidence that sex differences in cell-type populations exist for many tissues (Mank & Rideout, 2021). For example, differential rates of cell proliferation between males and females seem to underly the development of several sexually dimorphic ornamental traits, such as caudal fins in *Xiphophorus* (Powell et al., 2021; Schartl et al., 2021) and horns in rhinoceros beetles (Emlen et al., 2012). As such, it remains unclear to what extent are sex differences in expression a cause, as opposed to a consequence, of sexual dimorphism.

Sex-biased gene expression is often used to measure the footprint of sex-specific selection and sexual conflict within the genome. However, this approach has produced some discordant results (Grath & Parsch, 2016; Mank, 2017). Male-biased genes in many species tend to exhibit higher rates of evolution at both the coding sequence and the expression level (Khaitovich et al., 2005; Lichilín et al., 2021; Lipinska et al., 2015; Ranz et al., 2003; Sharma et al., 2014; Whittle & Extavour, 2019). While early work in *Drosophila melanogaster* has interpreted this as the result of stronger sexual selection acting in males (Proschel et al., 2006; Sawyer et al., 2007), studies in other species have found that such accelerated patterns of evolution are instead more consistent with relaxed constraint (Dapper & Wade, 2020; Djordjevic et al., 2022; Gershoni & Pietrokovski, 2014; Harrison et al., 2015; Sayadi et al., 2019), and experimental evolution results are mixed (Hollis et al., 2014; Li Richter & Hollis, 2021; Veltsos et al., 2017). Some of the discordance between these different studies may be due to the heterogeneity in tissue composition and varying allometric scaling across species. Distinguishing between sex-biased genes that are due to regulatory changes and those that are simply a consequence of developmental differences in cell-type abundance is important for these types of molecular evolutionary analyses as the former is expected to be more enriched for genes subject to sexual selection and influencing sexual dimorphism.

Single-cell transcriptomics (scRNA-seq) offers the possibility to avoid the challenges posed by measuring expression from a heterogeneous tissue by instead comparing expression level between samples across equivalent cell populations. Here we leverage these recent advances in scRNA-sequencing to determine to what extent sex-biased gene expression is the result of sex differences in cell-type abundance as opposed to regulatory differences between similar cells, and to assess how this impacts inferences of evolutionary divergence.

## Methods

### Tissue collection and dissociation

We sampled reproductively mature (>3 months of age) male and female guppies from our laboratory population. Sexual maturity in males was detected by the development of a gonopodium, a modified anal fin, and of coloration (Houde, 2019), and in females by the presence of a dark pigmented spot, the gravid spot, close to the anal pore. All fish were raised at a water temperature of 26°C with a 12:12 light:dark schedule and fed a daily diet of flake food and live *Artemia* brine shrimp. Fish were euthanized with a pH-neutralized MS222 solution and dissected immediately. We dissected liver, heart, tail skin (removing any muscle and scales), and gonad (testis in the case of males, and ovaries excluding embryos in the case of pregnant females) tissues and immediately placed them in phosphate-buffered saline (PBS) solution (Corning) on ice. We obtained three replicates for each tissue type and sex, and every replicate contained a nonoverlapping pool of five individuals in order to ensure sufficient material for single-cell dissociation, resulting in a total of 24 samples.

Tissues were incubated at 30°C in a solution of 4 mg/mL collagenase type I (Sigma-Aldrich) and 4 mM CaCl_2_, gently pipetting every 3 min using a wide-bore pipette tip, until digested. Dissociated tissues were then filter-strained using a Flowmi 40-μm cell strainer. We centrifuged the samples at 300 rcf for 3 min, removing the supernatant and resuspending the cell pellet in PBS containing 0.04% bovine serum albumin (BSA) (Corning). Samples were centrifuged and resuspended in PBS + 0.04% BSA twice and immediately placed on ice before further processing.

### Single-cell library preparation and sequencing

We mixed 10 μL of cell solution with 10 μL of the exclusion dye trypan blue 0.4% (Invitrogen) and estimated cell viability and concentration using a Countess II automated cell counter (ThermoFisher). An estimated 8,000 cells from each sample were then loaded onto individual lanes of a 10× Genomic Chromium Controller and barcoded 3ʹ single-cell libraries were prepared using the 10X Genomics Chromium Next GEM Single Cell 3’ kit v3.1 following the manufacturer’s instructions (#CG0000204). We assessed the quality and concentration of cDNA and of libraries using an Agilent 4200 TapeStation and the Agilent High Sensitivity D5000 ScreenTape system. Libraries were sequenced on an Illumina NovaSeq 6000 sequencer, with an average sequencing depth of 20,000 read pairs per cell.

### scRNA-seq data processing

We used CellRanger v5.0.1 with the “mkref” function (Zheng et al., 2017) to build a reference index using the Ensembl *P. reticulata* genome (GCA_000633615.2) and annotation (release-103) files. Using the CellRanger v5.0.1 “count” function, we then aligned sequencing reads from fastq files to the reference index, identified cell-associated barcodes, and extracted gene-by-cell count matrices. Data filtering and downstream analyses were performed using Seurat v4.1.0 (Satija et al., 2015) in R v4.0.5 (R Core Team, 2021). We filtered the count data by keeping genes expressed in at least three cells and cells with at least 100 expressed genes. Raw counts were then normalized and scaled for each sample to account for differences in sequencing depth per cell using the “SCTransfrom” function (Hafemeister & Satija, 2019). For each cell type of each tissue, we calculated and plotted Pearson correlations between the number of genes and the number of reads per cell, before and after normalization, using the “FeatureScatter” function in Seurat. We used the function “doubletFinder_v3” from the DoubletFinder v2.0.3 package (McGinnis et al., 2019) in R to identify and remove doublets, which are the result of random encapsulation of more than one cell within the single-cell bead as part of the microfluidics process.

### scRNA-seq data clustering

We performed the principal component analysis (PCA) using the “RunPCA” function and identified the significant PCs using the “ElbowPlot” function. We then constructed the nearest-neighbor graph with the “FindNeighbors” function and performed graph-based clustering with the “FindClusters” function. We used the clustree v0.4.4 package in R to guide the decision of the optimal resolution to choose for the clustering analysis (Zappia & Oshlack, 2018). Clusters were then visualized using uniform manifold approximation and projection (UMAP) embedding with “RunUMAP” (Supplementary Figure S1). For each tissue, we used the “FindAllMarkers” function in Seurat to identify differentially expressed genes for each cell cluster. We identified and annotated the different cell types within each tissue based on marker gene information from the Zebrafish Cell Landscape (Jiang et al., 2021; Wang et al., 2023) and other published work (Liu et al., 2022; Morrison et al., 2021; Qian et al., 2022) (Supplementary Figure S2).

### Differential cell-type abundance analysis

For each tissue type, we tested for significant differences between male and female samples in the abundance of the identified cell types. We first quantified the number of cells assigned to each cell type and sample. We then merged counts for each sex and calculated the proportion of cells of each cell type out of the total number of cells. We added 1e-10 to each proportion value to avoid infinitely high numbers associated with log_2_ 0 and calculated the female-to-male fold change (FC) in cell-type abundance as log_2_ (female proportion/male proportion). To identify significant differences in cell-type abundance between the sexes, we computed two-proportion *z*-tests (*p* < .01) with the “prop.test” function in R.

### Sex-biased gene expression analysis

To identify sex-biased genes at the cell level, for each identified cell type, we aggregated raw counts across all cells to the sample level using the “aggregate.Matrix” function in R. We then used DESeq2 to normalize the count data accounting for differences in library size between samples and applying a log2-transformation with the “rlog” function (Love et al., 2014). Lastly, we performed differential expression analysis using the “DESeq” function. Sex-biased genes were called based on |log_2_ FC| ≥ 1 and a false discovery rate (FDR) adjusted *p*-value < .05 to correct for multiple testing. To identify sex-biased genes at the bulk level, we followed the same steps described above, but instead aggregated counts across all cells from all clusters together to obtain a single expression value for each gene and sample.

In addition, we tested the extent to which expression estimates from bulked single-cell RNA-seq data correlate with those from bulk RNA-seq data. For this, we analyzed publicly available bulk RNA-seq data from adult male and female gonad (Sharma et al., 2014) and male skin tissue (NCBI SRA PRJDB11269). We used HISAT2 v2.0.4 (Kim et al., 2015) to map reads to the *P. reticulata* genome keeping paired (--no-mixed) and concordant (--no-discordant) alignments only. We estimated gene expression by extracting read counts for each gene using HTSeq-count (Anders et al., 2015). We log2-transformed counts and normalized with regards to library size using the “rlog” function in DESeq2. We performed Spearman correlations of gene expression with the “cor.test” function in R.

### Rates of coding-sequence evolution analysis

We obtained coding sequences from the outgroup species *Gambusia affinis* (ASM309773v1), *Xiphophorus maculatus* (Xipmac4.4.2), and *Oryzias latipes* (MEDAKA1) from Ensembl 104 and extracted the longest isoform for each gene. We used reciprocal BLASTn v2.7.1 (Altschul et al., 1990) with an *e*-value cutoff of 10e-10 and a minimum percentage identity of 30% to determine orthology across the *P. reticulata* genes and outgroup sequences. For genes with multiple blast hits, we chose the top hit based on the highest BLAST score.

We used *O. latipes* (MEDAKA1) protein-coding sequences from Ensembl 104 and BLASTx v2.3.0 with an *e*-value cutoff of 10e-10 and a minimum percentage identity of 30% to obtain open reading frames. We excluded orthogroups without BLASTx hits or valid protein-coding sequences. We aligned orthologous gene sequences with PRANK v170427 (Loytynoja & Goldman, 2008) and filtered alignments to remove gaps. We also masked poorly aligned and error-rich regions with SWAMP (Harrison et al., 2014) with a threshold of 4 misalignments in a window size of 5 bp and a minimum sequence length of 100 bp.

To obtain divergence estimates, we used the branch model (model = 2, nssites = 0) in the CODEML package in PAML v4.8 (Yang, 2007). Genes with *d*_S_ > 2 were removed from subsequent analyses to avoid inaccurate divergence estimates due to mutation saturation and double hits (Axelsson et al., 2008). We divided genes into different sex-bias categories (see Figure 3) and extracted the number of nonsynonymous (*D*_N_) and synonymous substitutions (*D*_S_) and the number of nonsynonymous (*N*) and synonymous (*S*) sites. For each group of genes, we then calculated the mean rate of nonsynonymous substitutions (*d*_*N*_) and mean rate of synonymous substitutions (*d*_*S*_) as the number of substitutions across all genes divided by the number of sites (*d*_*N*_ = *D*_*N*_/*N*; *d*_*S*_ = *D*_*S*_/*S*). We used bootstrapping with 1,000 replicates to determine the 95% confidence intervals for divergence estimates in each gene group and tested for differences in *d*_*N*_, *d*_*S*_, and *d*_*N*_/*d*_*S*_ estimates between the different gene groups based on 1,000 replicates permutation tests.

## Results

We generated 24 scRNA-seq data sets from skin, heart, liver, and gonad tissue from adult male and female guppies, with three replicates for each sex and tissue. Guppies are highly sexually dimorphic, displaying sex differences in size (Houde, 2019), sexual ornaments (Reznick & Endler, 1982), life history (Reznick & Endler, 1982), and behavior (Reznick, 1989), among other traits, and we chose these four tissues to reflect a range of phenotypic dimorphism. Following quality control and filtering (see Methods), we recovered between 503–3,747, 1,163–8,966, 4,985–6,835, and 1,492–7,973 cells (Supplementary Table S1) and 9,275, 9,818, 11,540, and 13,617 genes expressed in skin, heart, liver, and gonad tissues, respectively (Supplementary Table S2). Following UMAP dimensionality reduction and marker-based annotation, we identified 13, 11, 9, and 8 distinctly expressed cell clusters for skin, heart, liver, and gonad, respectively (Supplementary Figures S1 and S2). These clusters are representative of most major cell types found in these tissues (Jiang et al., 2021; Wang et al., 2023).

### Sex differences in cell-type abundance across tissues

**Figure 1.**
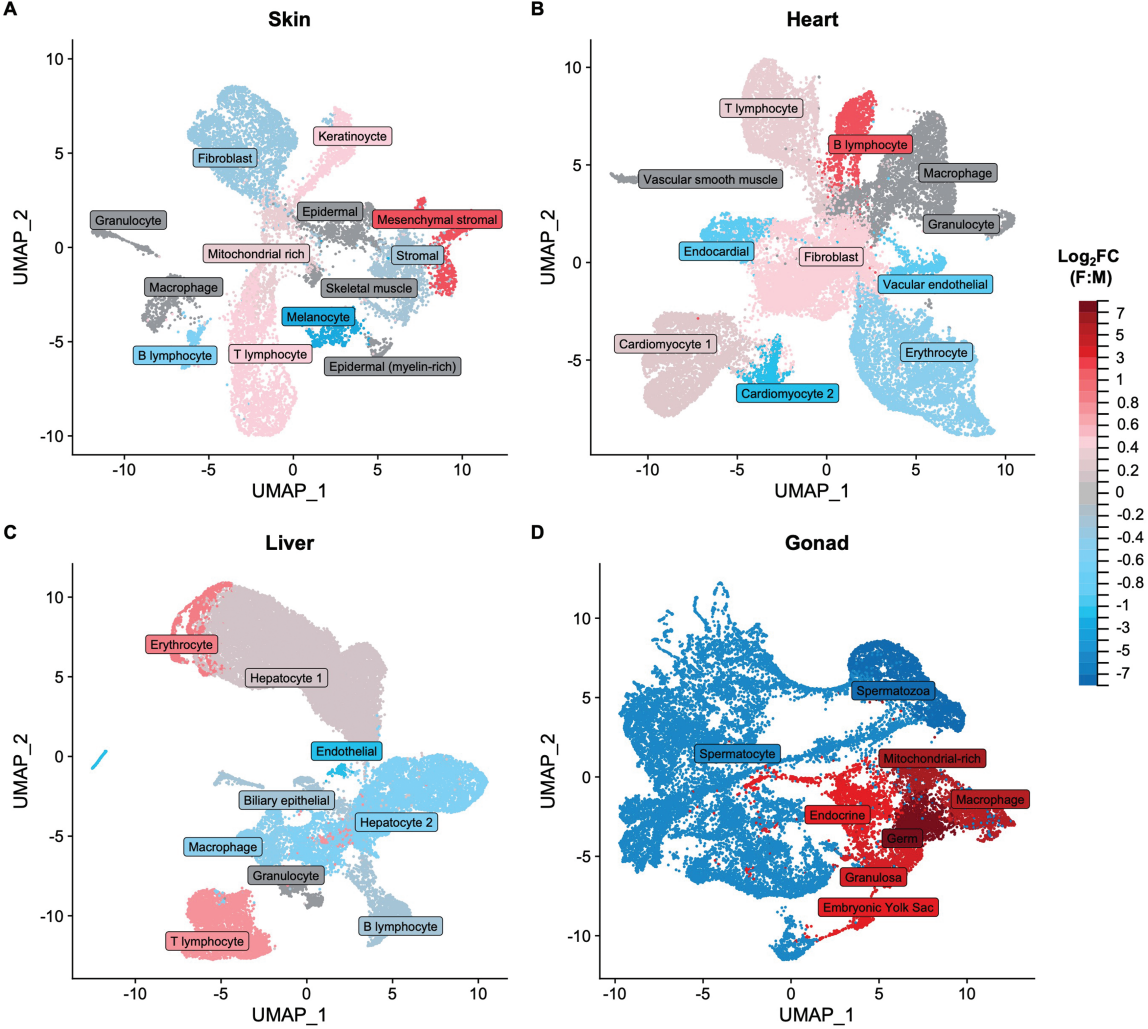
Differential cell-type abundance between males and females for skin (A), heart (B), liver (C), and gonad (D) tissues. Cell types that are significantly more abundant in females are shown in red, those that are significantly male-biased in abundance are shown in blue, while unbiased cell types are in gray. Significance based on two-proportions *z*-tests (*p* < .01).

We examined each tissue for differences in cell composition between the sexes based on the estimated abundances of each of the identified cell types. We found significant sex differences (*p* < .01, two-proportion *z*-tests) in abundance for many cell populations (Figure 1; Supplementary Table S3), the most extreme differences being present in the reproductive tissue, where most of the cell types are sex limited (Figure 1D, Supplementary Figure S3). Notably, the vast majority of cells in the male gonad are related to sperm, while the female gonad represents a broader mix of gametic and somatic cells (Supplementary Figure S3). However, we observe a range of dimorphism in cell composition among somatic tissues as well. The liver plays a critical role in several physiological processes, including hormonal regulation, metabolism, digestion, and immune response (Bruslé & Anadon, 1996), and shows marked sex differences (Marcos et al., 2015; Ullah et al., 2021). Consistent with the more sexually dimorphic nature of this somatic tissue, we found extensive sex differences in cellular composition, with all but one of the identified cell types showing a significant sex-bias in abundance (Figure 1C). Although the heart and skin tissues are less sexually dimorphic compared with the liver, we also recovered sex differences in abundance for more than half of the cell populations in these two tissues (Figure 1A and B). Markedly, guppies exhibit striking sexual dimorphism in skin pigmentation, with male-specific ornamental color patterns (Reznick & Endler, 1982), which we also find reflected here through the strong male-biased abundance of melanocytes in males (Figure 1).

### Sex-biased gene expression at bulk and cell level

Dropout events are a well-known challenge with scRNA-seq data, where the prevalence of genes with nonbiological zero expression measurements can confound gene expression analyses. We employed a normalization and variance stabilization approach to reduce the effect of technical noise on estimates of gene expression (Hafemeister & Satija, 2019). Indeed, for most cell types across each tissue, we find a significantly decreased correlation between the expression level of a gene and the total sequencing depth of a cell following normalization (Supplementary Figure S4). This indicates that our gene expression analyses should not be influenced by variation in sequencing depth and that differences in gene expression across cells primarily reflect biological heterogeneity (Hafemeister & Satija, 2019).

**Figure 2.**
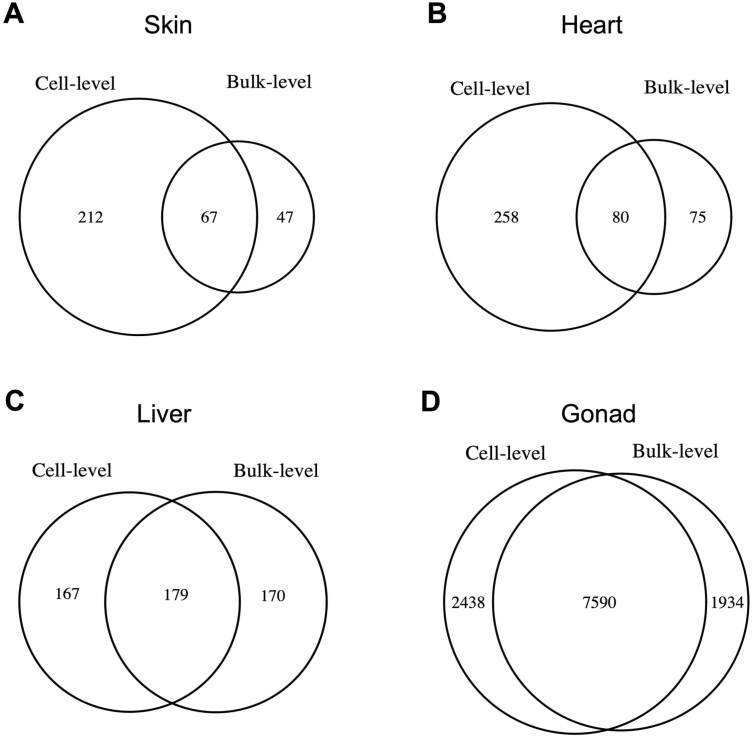
Number of genes showing sex-biased gene expression at the cell and bulk level in skin (A), heart (B), liver (C), and gonad (D) tissues. Numbers at the “Cell-level” represent the union of genes identified as sex-biased across all the different cell types. Significant differential expression between the sexes was based on |log_2_ FC| ≥ 1 and an FDR-corrected *p*-value < .05.

For each tissue, we aggregated expression measures across all cells to obtain a single value for each gene and sample, thus reflecting expression at the bulk tissue level. Using the bulked scRNA-seq data, we identified genes with a sex-biased expression profile (|log_2_ fold change| ≥ 1, FDR-corrected *p*-value < .05). Consistent with previous estimates in guppies (Sharma et al., 2014), we find that, at the bulk level, male and female gonads exhibit strong patterns of sex-biased gene expression, with 9,524 genes identified as differentially expressed, representing 70% of all genes expressed in the gonads (Figure 2D, Supplementary Table S2). Top male-biased genes included those encoding for spermatogenesis processes (*march11*, *morn3*, *spata18*, *spatc1l*), ciliary- and flagellar-associated proteins (*cfap52*, *ropn1l*, *tekt1*), male-specific development (*dmrt1*), and calcium-binding proteins (*efcab2*), while female-biased genes encoded for, among others, zona pellucida glicoproteins (*zp2l2*, *zp3f.1*, *zp3d.2*), oocyte-specific proteins (*zar1*, *zar1l*), and ovarian folliculogenesis processes (*gdf9*, *cx43.4*, *pcdh18a*) (Supplementary Table S2).

Given their sex-specific roles, reproductive organs are by far the most transcriptionally dimorphic tissues; however, there is substantial variation in sex differences in expression across somatic tissues as well (Khodursky et al., 2020; Ma et al., 2018; Mank et al., 2007; Oliva et al., 2020; Yang et al., 2006), which we also observe here. The liver was the most transcriptionally dimorphic somatic tissue, with 349 sex-biased genes, more than twice as many compared with the skin (114) and heart (155) tissues (Figure 2A–C). The liver has been shown to be one of the somatic tissues with the most sex-biased gene expression profiles in other fish species (Qiao et al., 2016; Rose et al., 2015; Taboada et al., 2012; Zheng et al., 2013). In fish and other oviparous vertebrates, the liver carries an important role in the vitellogenesis process of egg yolk protein synthesis, transport, and uptake in the maturing oocyte (Arukwe & Goksøyr, 2003; Hara et al., 2016), and, in line with this, we also find vitellogenins (*vtg1*, *vtg2*, *vtg3*) to be some of the top female-biased genes in the liver (Supplementary Table S2). While skin and heart tissues express fewer sex-biased genes, we also note highly male-biased genes in skin with a role in pigmentation (*fhl2a*, *pnp4a*).

To test whether bulked scRNA-seq expression data correlates with bulk RNA-seq data, we analyzed publicly available bulk RNA-seq data for adult male and female gonad and male skin tissue. Unfortunately, bulk data for the other tissues were not available. We found a strong correlation in gene expression estimates between the bulk RNA-seq and the bulk scRNA-seq datasets for both male gonad and male skin tissues (Supplementary Figure S5). Multiple factors, such as technical variation and differences in sample preparation, can explain the lack of correlation for the female gonad tissue. We observe a very strong correlation between the three bulk scRNA-seq female gonad samples (Supplementary Figure S5). However, there was only one bulk RNA-seq female gonad sample available which prevented us from testing for the effects of technical noise on the correlation analysis. Nevertheless, previous work in other systems has shown a strong correlation between expression estimates from bulk RNA-seq and bulk scRNA-seq data across various tissues (Karlsson et al., 2021; Marinov et al., 2014; Trapnell et al., 2014).

We next compared patterns of differential gene expression between males and females at the whole tissue level with those at the cell level. As such, in addition to the aggregated bulk-level expression, for each identified cell type we aggregated expression data across all cells to identify sex-biased genes in each cell population. Overall, we discovered more sex-biased genes at the cell level than at the bulk level in all tissues, except for liver. However, what is even more striking is that across the somatic tissues between 49% and 77% of the identified sex-biased genes at the cell level have unbiased expression profiles at the bulk level (Figure 2). By contrast, in the gonad, only about 24% of the differentially expressed genes at the cell level show no sex differences in expression at the bulk level. These results indicate that, outside of the reproductive tissues, differential gene expression analyses based on bulk RNA-sequencing data are limited in their ability to comprehensively identify sex-biased genes. Nonisometric-scaling relationships between cellular subcomponents of a tissue may cause patterns of differential expression in low abundance cell types to be concealed when expression is aggregated across various cell types in standard bulk RNA-sequencing experiments, thus creating a false-negative inference of sex-biased gene expression.

Moreover, in all somatic tissues, many genes are characterized as sex-biased at the bulk level, but not at the cellular level. We hypothesized that these differential expression patterns are not due to regulatory rewiring within cell types but instead a consequence of differences in cell-type abundance between males and females. Indeed, these genes tend to have a significantly lower magnitude of expression fold change (Supplementary Figure S6) and are more highly expressed in cell types that exhibit significant sex differences in abundance (Supplementary Figure S7). In skin, genes that are female-biased at the bulk level only are predominantly expressed in the mesenchymal stromal cell population which is more abundant in females. Similarly in liver, genes that are female- and male-biased in expression at the bulk level but not at the cell level have a significantly higher expression in the female-abundant T lymphocytes and the male-abundant endothelial cells, respectively. Although in the gonad a much smaller percentage of genes are sex-biased only at the bulk level, these too appear to be the result of sex differences in cell-type abundance (Supplementary Figure S7). These results show that heterogeneity in cellular scaling relationships, due to developmental differences in cell proliferation, between males and females can influence perceived patterns of differential gene expression in the absence of regulatory changes.

### Rates of evolution of sex-biased genes

We tested whether the cause of sex-biased gene expression influences estimates of coding-sequence evolution. We hypothesized that sex-biased genes that are due to sex differences in cell-type abundance, and thus a consequence of sex-specific developmental trajectories, may not be direct targets of sex-specific selection and may not show elevated rates of sequence divergence compared with unbiased genes. By contrast, loci exhibiting regulatory differences between males and females are more likely to underly sexually dimorphic phenotypes and would be expected to show stronger signatures of sex-specific selection, if indeed these genes as a class are more likely to be the focus of sexual selection.

**Figure 3.**
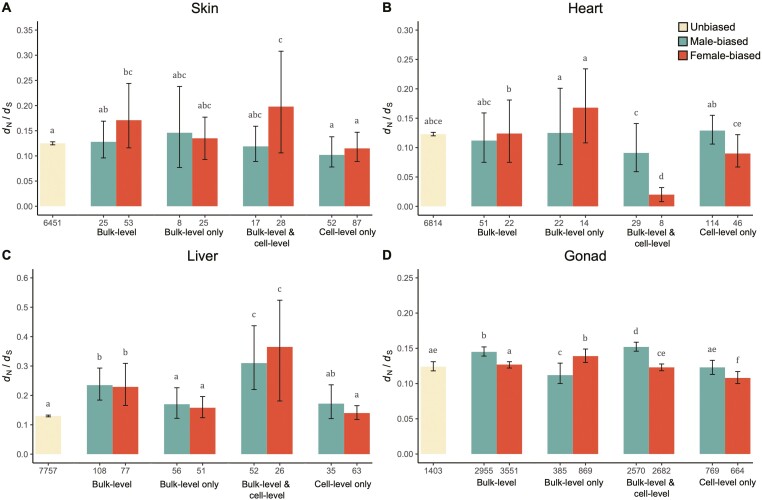
Rates of coding-sequence evolution (*d*_N_/*d*_S_) for genes in skin (A), heart (B), liver (C), and gonad (D). Shown are estimates of divergence for genes that are unbiased at both the bulk level and cell level (yellow), all genes identified as male-biased (green) and female-biased (orange) at the bulk level (Bulk-level), genes with a male-biased and female-biased expression at the bulk level but that are unbiased in every cell type within that tissue (Bulk-level only), genes that show a sex-biased expression profile both at the bulk level and in at least one of the identified cell types in that tissue (Bulk-level and Cell-level), and sex-biased genes at the cell level but not at the bulk level (Cell-level only). Numbers on the x-axis represent the number of genes in each group. Letters above bars indicate pairwise significance between groups based on 1,000 replicates permutations, where bars that share a letter are not significantly different, while bars with no overlapping letters are significantly different (*p* < .05).

When considering all the differentially expressed genes at the bulk level, we found that male-biased genes in liver and gonad tissues and female-biased genes in skin, liver, and gonad tissues evolve significantly faster than unbiased genes (Figure 3), owing to higher rates of nonsynonymous substitutions (Supplementary Table S4). To further understand what drives these elevated divergence patterns at the bulk level, we split genes into those that are sex-biased at the bulk level but unbiased at the cell level, which are more likely to result from sex differences in cell-type abundance (Supplementary Figure S7), and those that are sex-biased at both the bulk and cell level, and which are due to regulatory sex differences. We found that the high divergence rates observed at the bulk level are driven by the subset of genes that are sex-biased at both the bulk and the cell level, as these genes exhibited an even more accelerated rate of evolution (Figure 3) compared to unbiased genes. These loci are more likely to be underlying sexually dimorphic phenotypes as they also exhibit elevated log_2_FC estimates (Supplementary Figure S6). On the other hand, the subset of genes that are sex-biased at the bulk level only, and thus, the outcome of differential tissue composition between the sexes, showed similar rates of coding-sequence evolution to unbiased genes, further suggesting that they are not the main targets of selection. An exception to this may be highly differentially expressed genes in the gonad that have important reproductive functions, such as sperm-specific genes, and which would therefore be subject to sexual selection. In general, however, we observe that false-positive differentially expressed genes can obscure true patterns of evolutionary divergence for sex-biased genes at the bulk level.

Previous work has noted that the level of sequence divergence increases with the degree of sex-biased gene expression (Djordjevic et al., 2022; Harrison et al., 2015; Lichilín et al., 2021). To test this, we split sex-biased genes from the gonad tissue into different groups based on their level of sex-bias. We also find that highly male-biased and female-biased genes exhibit greater divergence than lowly male-biased and female-biased genes (Supplementary Figure S8). Considering that we observe a higher expression fold change for genes that are sex-biased at both the bulk and the cell level (Supplementary Figure S6), this suggests that the accelerated rates of sequence divergence for this group of genes may at least in part be due to their more extreme sex-bias in expression. However, the small number of sex-biased genes for which we could estimate rates of coding-sequence evolution prevented us from investigating this further in the somatic tissues.

## Discussion

To determine the relative role of regulatory versus developmental differences underlying sex-biased gene expression, we used single-cell transcriptomic data from multiple somatic and reproductive tissues of male and female guppies, a species that exhibits extensive phenotypic sexual dimorphism. Sex-biased genes can be a cause of sexual dimorphism, as is often assumed in studies that use them to study the molecular genetic footprint of sexual selection and sexual conflict (e.g., Harrison et al., 2015; Mank, 2017; Sayadi et al., 2019). Alternatively, sex-biased genes may be a consequence of developmental differences in cell proliferation between the sexes that result in differences in cell-type abundances. Sex differences in cell populations are known to exist in many tissues (Mank & Rideout, 2021), and differential rates of cell proliferation underly the development of several sexually dimorphic ornamental traits (Emlen et al., 2012; Powell et al., 2021; Schartl et al., 2021). The question of whether sex-biased genes are a cause or consequence of sexual dimorphism is critical, as the former might be subject to differences in sex-specific selection and therefore useful in studies of sexual selection, sexual dimorphism, and sexual conflict, while the latter are largely a consequence of differences in developmental programming.

Our analysis of gene expression at single-cell resolution illustrates that cellular heterogeneity within tissues and allometric scaling differences of cell types between males and females can have a major influence on inferred patterns of sex-biased gene expression. Such scaling effects can generate both false-negative detection of differentially expressed genes, which may preponderantly concern genes that are expressed in low abundance cell types, as well as false-positive patterns of differential gene regulation, as is the case for genes that are sex-biased at the bulk tissue level but not at the cell level. These false-positive results have the potential to affect inferences on the evolution of sex-biased gene expression in several ways.

Allometry can be a confounding factor in estimates of rates of coding-sequence evolution for sex-biased genes, as we show that false-positive sex-biased genes diminish the signal of elevated rates of divergence for both male- and female-biased genes. Variation in the fraction of genes that are erroneously classified as sex-biased due to sex differences in tissue composition may explain some of the discordance in patterns of rapid rates of coding-sequence evolution and sex-specific selection observed across studies (Harrison et al., 2015; Khaitovich et al., 2005; Lipinska et al., 2015; Mank et al., 2010; Ranz et al., 2003; Whittle & Johannesson, 2013). Sex differences in gene co-expression networks are thought to contribute to sexually dimorphic phenotypes and potentially alleviate sexually antagonistic selection (Lopes-Ramos et al., 2020; Rago et al., 2020; Sutherland et al., 2019). Yet fundamental differences in gene regulatory networks can exist between cell types and variation in cell-type abundance between the sexes can also affect gene co-expression measurements (Ribeiro et al., 2022).

The degree of sex differences in cell-type abundance likely varies across species as a function of phenotypic sexual dimorphism, and this can influence patterns of rapid turnover of sex-bias across species. We observed large differences in cell-type abundance between the sexes (Supplementary Table S3, Figure 1, Supplementary Figure S3), with the liver showing the least overall fold change in cell-type abundance, moderate levels in the skin and heart, and the greatest observed in the gonad. In some cases, cell-type abundance differences were consistent with visible phenotypic differences, such as the male-bias in melanocytes in male skin, as might be expected from male coloration. However, many cell types with sex differences might not necessarily be predicted from phenotypic differences, such as the male-bias in heart cardiomyocytes, or the female-bias in skin mesenchymal stromal cells. Identifying distinct cell populations for non-model organisms relies largely on databases of cell-type-specific marker genes from model species, which may be distantly related. While our analysis did not exhaustively identify all the different cell subpopulations, male and female homologous cell clusters were annotated in the same way, and we were able to recover all the major cell types expected within each tissue (Jiang et al., 2021; Wang et al., 2023).

Notably, spermatocytes and spermatozoa combined make up 97% of male gonad cells. In contrast, the female gonad is comprised of only 20% germ cells, with the remainder a mix of somatic cells. These patterns are consistent with previous findings in zebrafish (Jiang et al., 2021) and point to the fact that bulk comparisons between male and female gonads, or whole bodies where the majority of expression differences are due to the gonad (Parisi et al., 2004), are in practice comparing expression related to sperm in males with a range of cell functions in females. Male-biased genes identified from whole-organism or bulk gonad preparations in many species show rapid rates of protein evolution (Ellegren & Parsch, 2007; Parsch & Ellegren, 2013; Ranz et al., 2003) and exhibit high rates of turnover (Harrison et al., 2015; Khodursky et al., 2020; Papa et al., 2017; Whittle & Extavour, 2019; Zhang et al., 2007), both of which might be expected from sexual selection predictions. Indeed, in our bulk analysis, male-biased genes in the gonad show elevated rates of evolution (Figure 3D). However, it is important to note that genes with restricted expression (e.g., restricted to sperm) often experience fewer adaptive constraints (Yannai et al., 2005) than those with broader expression. Indeed, when comparing genes that are sex-biased within gonad cell types, rather than those that differ as a result of cell-type abundance, male-biased genes in the gonad evolve at the same rate as unbiased genes (Figure 3D).

Overall, our results suggest that sex differences in cell-type abundance scale with visible sexual dimorphism, suggesting that bulk RNA-seq approaches may be increasingly problematic in more dimorphic species. Single-cell transcriptomics, such as that employed here, offer a promising way to correct for any potential false inferences of differential gene expression and patterns of sequence divergence that are associated with standard bulk-level sequencing of heterogeneous tissue samples. However, although scRNA-sequencing approaches are increasingly employed for disentangling the role of regulatory changes in the evolution of intra- and interspecific phenotypic variation (Fuess & Bolnick, 2021; Murat et al., 2023), the associated costs are still substantially higher compared with standard bulk RNA-sequencing and many analytical challenges remain (Alfieri et al., 2022). Cell isolation protocols, especially for non-model organisms, are nontrivial as cell physiology may differ across tissues and species, and often require organism-specific technical knowledge to avoid cell death or bias in expression profiles (Reichard & Asosingh, 2019; Wang et al., 2021). Incomplete or poorly annotated genomes can additionally limit the identification of cell identities and gene expression patterns (Healey et al., 2022), although several *k*-mer-based methods have been developed to aid with de novo transcriptome assembly and identification of cell populations in cases where a reference genome is lacking (Nip et al., 2020; Sun et al., 2021). Compared with bulk RNA-seq data, scRNA-seq data are also affected by a greater sparsity, where a high proportion of genes have zero expression measurements within cells (Jiang et al., 2022). This sparsity is often due to cell-to-cell variation in technical factors, such as the number of molecules detected per cell and stochastic sampling, which can confound results (Stegle et al., 2015). However, normalization and variance stabilization approaches, such as the one employed here, can reduce the effect of technical noise on estimates of gene expression (Hafemeister & Satija, 2019). Moreover, performing differential gene expression analyses on aggregate cell data within each cell type, rather than on individual cells, reduces the number of zeroes in the data and offers better performance especially for lowly expressed genes (Squair et al., 2021).

In the absence of scRNA-sequencing data or information regarding the cellular composition of tissues, we suggest that adopting more stringent fold change thresholds for calling differentially expressed genes has the potential to substantially reduce the false-positive rates associated with bulk-level sequencing of heterogeneous tissues. Although a few studies have accounted for increasing fold change thresholds when studying the evolution of differential gene expression (Darolti et al., 2018; Djordjevic et al., 2022; Harrison et al., 2015; Ma et al., 2018, 2020; Mariani et al., 2003; Perry et al., 2014), others rely on a log_2_ fold change of 1 or lower, or on statistical significance alone. However, our results from bulk-level analyses show that such lowered thresholds can result in the inclusion of many sex-biased genes that are due to differences in cell-type abundance between males and females (Supplementary Figure S6). Although this attempt to reduce false-positive effects may also inadvertently remove some true-positive sex-biased genes, strong patterns of transcriptional dimorphism will remain unaffected. This approach would be particularly recommended for studies in which estimating additional tissue scaling parameters is limited due to the small size of the organism or difficulty in precise tissue dissection. Alternatively, deconvolution methods may also prove useful (Aguirre-Gamboa et al., 2020; Monaco et al., 2019).

Taken together, our findings offer an important insight into the effect of allometry and heterogeneity in tissue composition on inferred patterns of sex-biased gene expression and demonstrate the power of single-cell RNA-sequencing in differentiating sex-biased genes that stem from regulatory rewiring between males and females from those that are due to differences in cell-type abundance resulting from sex-specific developmental trajectories.

## Supplementary Material

qrad013_suppl_Supplementary_Material

qrad013_suppl_Supplementary_Table_S2

qrad013_suppl_Supplementary_Table_S4

## Data Availability

scRNA-sequencing reads have been deposited to the NCBI Short Read Archive (BioProject ID PRJNA902547). Scripts used for data processing and analysis are available on GitHub (https://github.com/manklab/Darolti_and_Mank_2022_Guppy_SingleCellExpression) and are archived on Zenodo (https://doi.org/10.5281/zenodo.7788730).
