# Supplementary material for: Sex-biased gene expression at single-cell resolution: cause and consequence of sexual dimorphism
Source: Evol Lett. 2023 Apr 14;7(3):148–56. doi: 10.1093/evlett/qrad013 (PMC10210449; doi:10.1093/evlett/qrad013)
Supplement: qrad013_suppl_Supplementary_Material [file qrad013_suppl_supplementary_material.pdf]

Supplementary Information for *Sex-biased gene expression at single-cell resolution: Cause and consequence of sexual dimorphism*

Iulia Darolti[1,2] & Judith E. Mank[1]

1 Department of Zoology and Biodiversity Research Centre, University of British Columbia, British Columbia, Canada

2 Department of Ecology and Evolution, University of Lausanne, Lausanne, Switzerland

Corresponding author: Iulia Darolti, e-mail: iulia.darolti@unil.ch

Fig. S1. UMAP plot showing cell clustering of scRNA-seq datasets and identified cell populations in skin (A), heart (B), liver (C) and gonad (D).

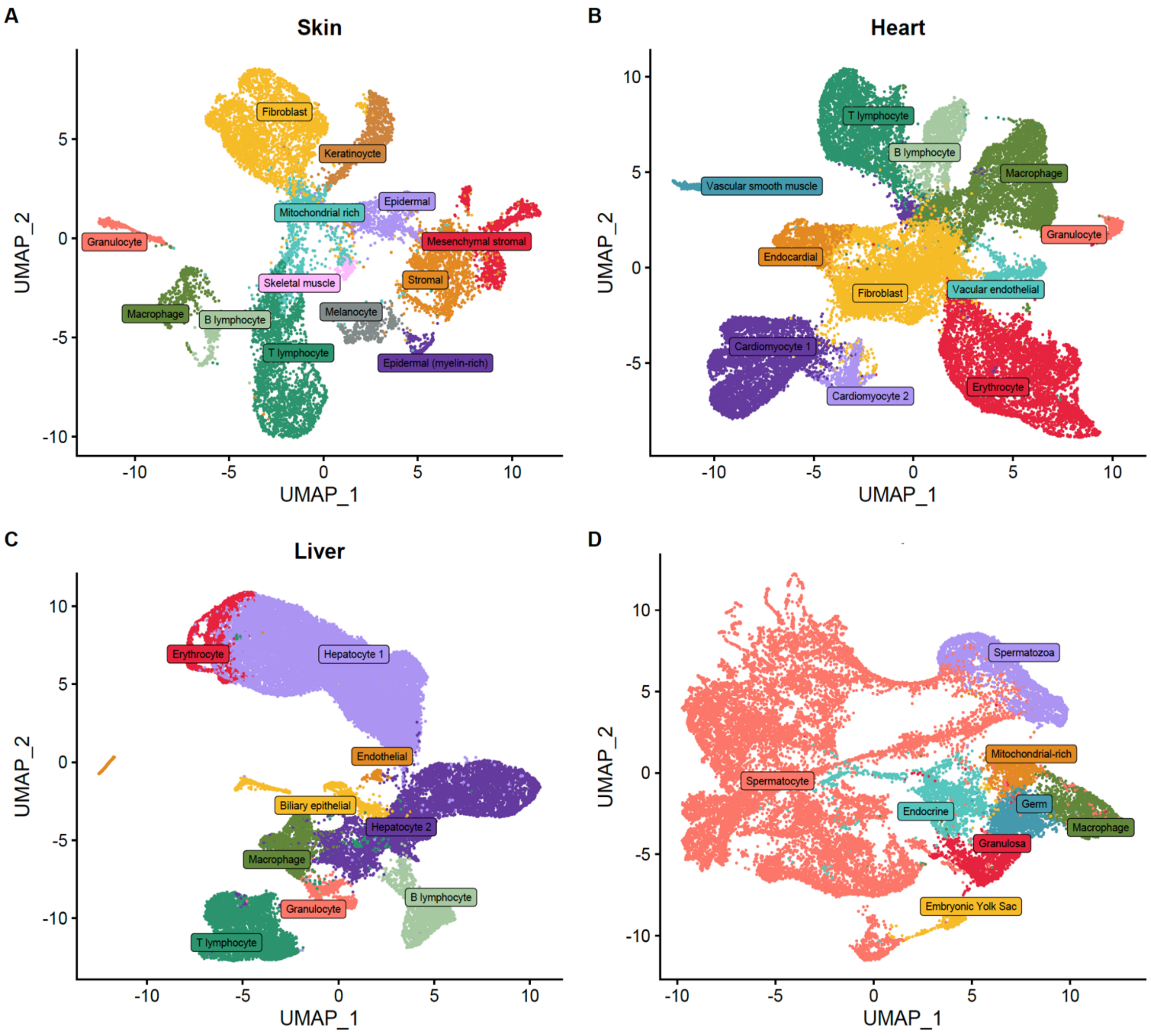

Fig. S2. Marker gene expression across identified cell types of each tissue.

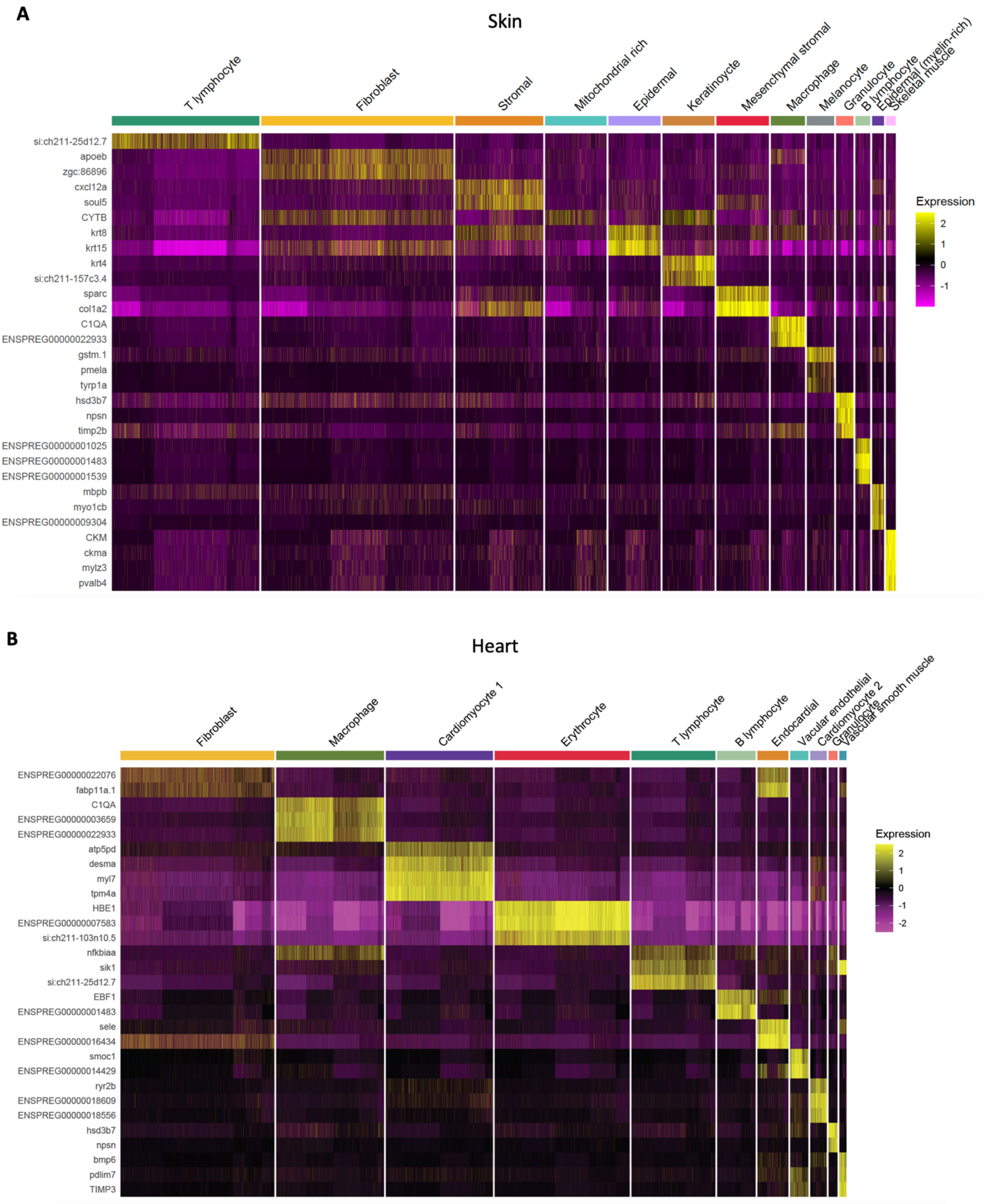

C

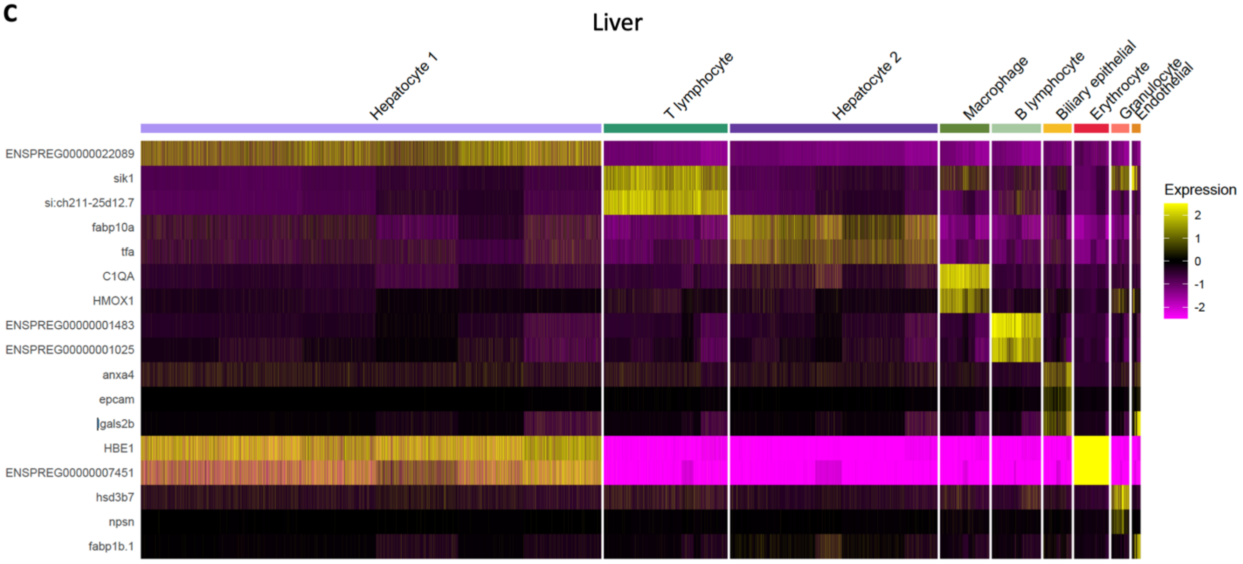
Liver
Hepatocyte 1
T lymphocyte
Hepatocyte 2
Macrophage
B lymphocyte
Biliary epithelial
Erythrocyte
Granulocyte
Endothelial
ENSPREG00000022089
sik1
si:ch211-25d12.7
fabp10a
tfa
C1QA
HMOX1
ENSPREG00000001483
ENSPREG00000001025
anxa4
epcam
lgals2b
HBE1
ENSPREG00000007451
hsd3b7
npsn
fabp1b.1
Expression
2
1
0
-1
-2

D

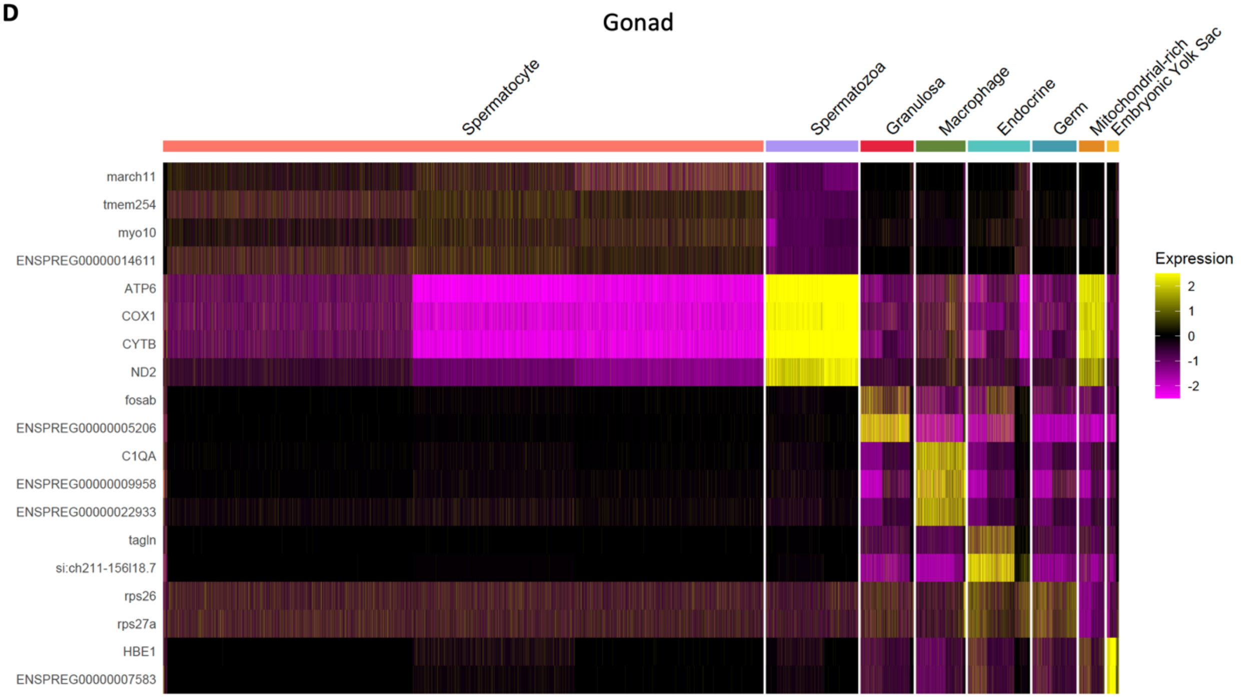
Gonad
Spermatocyte
Spermatozoa
Granulosa
Macrophage
Endocrine
Germ
Mitochondrial-rich
Embryonic Yolk Sac
march11
tmem254
myo10
ENSPREG00000014611
ATP6
COX1
CYTB
ND2
fosab
ENSPREG00000005206
C1QA
ENSPREG00000009958
ENSPREG00000022933
tagln
si:ch211-156l18.7
rps26
rps27a
HBE1
ENSPREG00000007583
Expression
2
1
0
-1
-2

Fig. S3. Average male and female proportion of each identified cell type within each tissue.

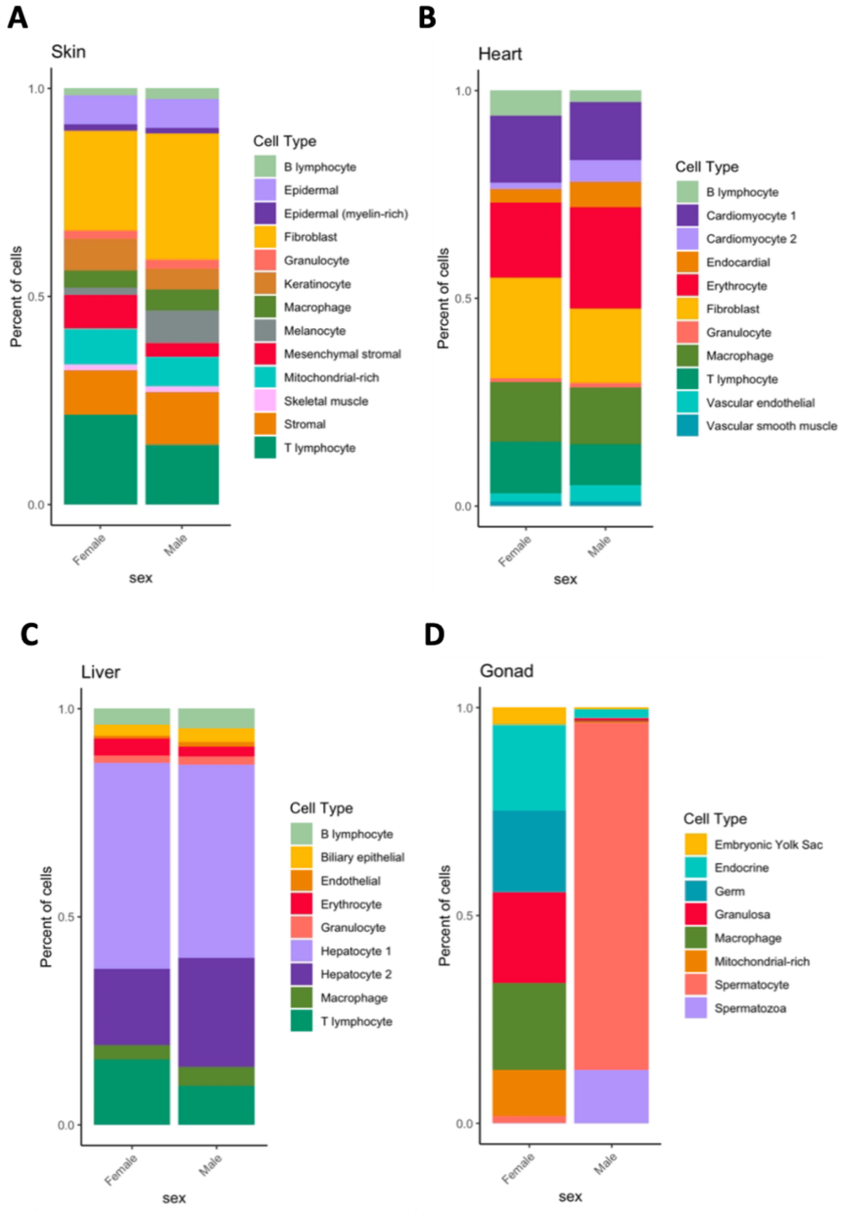

Fig. S4. Correlation between the number of reads and the number of genes per cell before (left) and after (right) normalization and variance stabilization. Average Pearson correlations across all cell types of each tissue are shown above each plot.

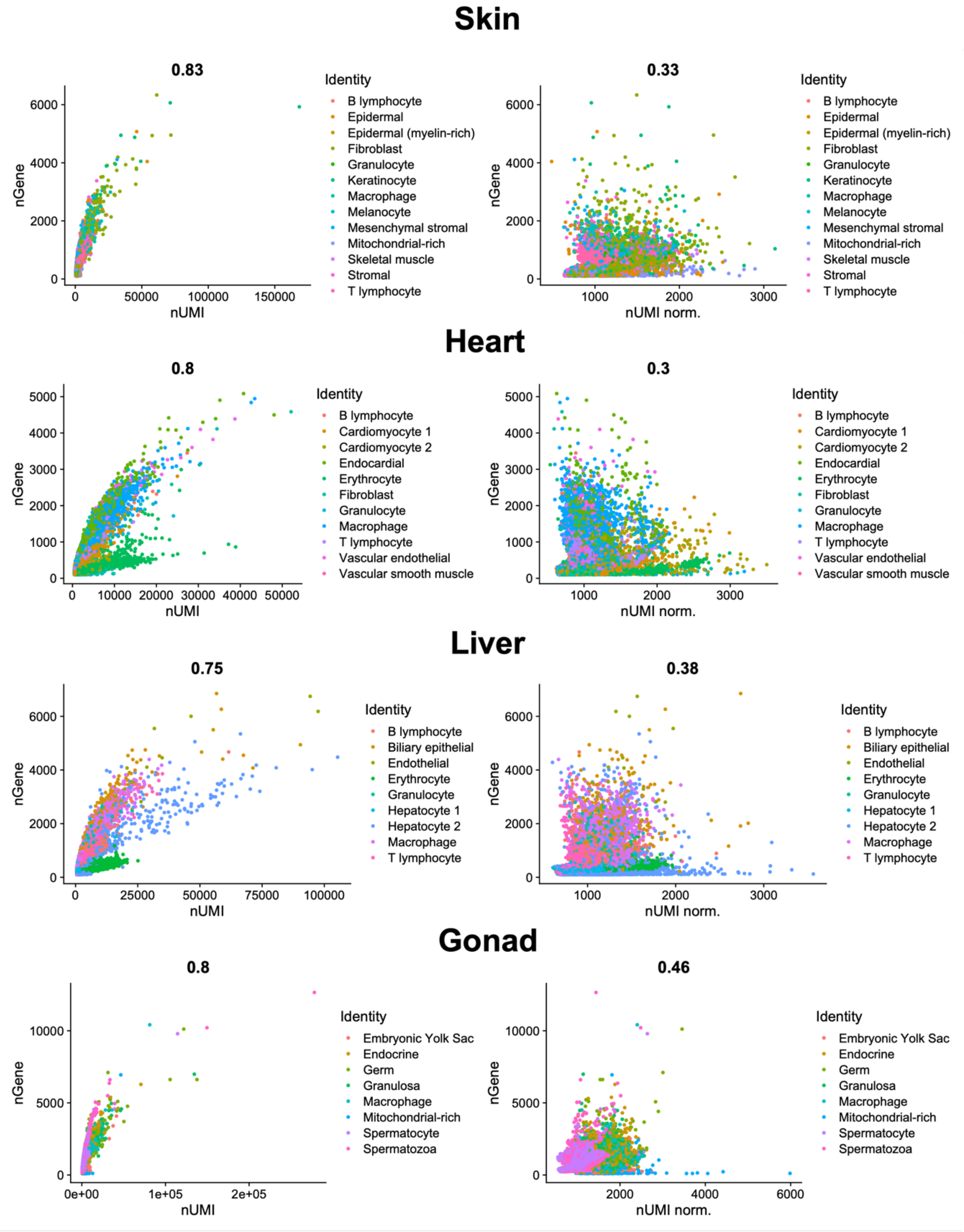

Fig. S5. Spearman correlations for gene expression estimates.

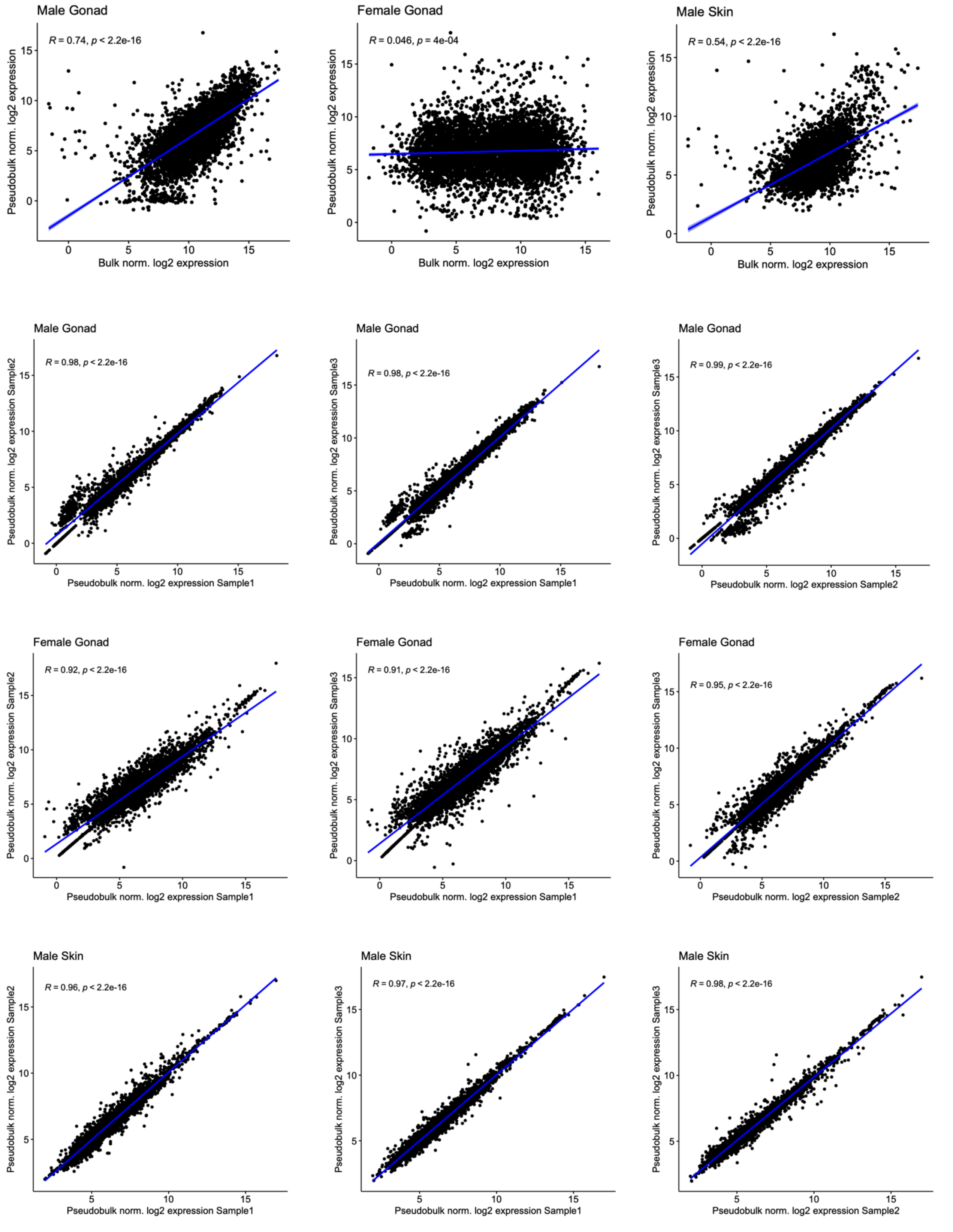

Fig. S6. Magnitude of $\log_2$ fold change in expression for female-biased (orange) and male-biased (green) genes. Significance values are based on comparisons between genes with the same sex-bias direction, calculated using paired Wilcoxon's signed-rank tests (*** $p < 0.001$).

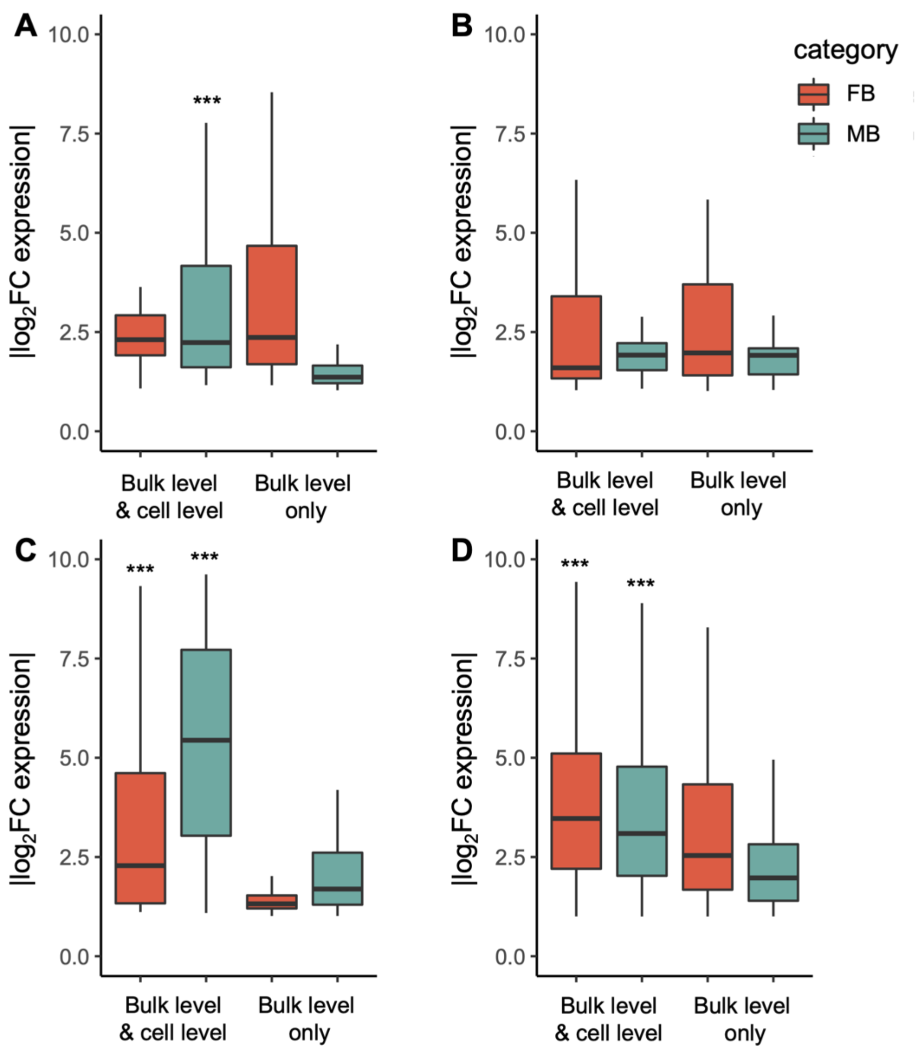

Fig. S7. Expression (log$_2$ CPM) for each cell type within each tissue for genes identified as male-biased (green) and female-biased (orange) at the bulk level only. Significance values are calculated using paired Wilcoxon's signed-rank tests (*** $p < 0.001$).

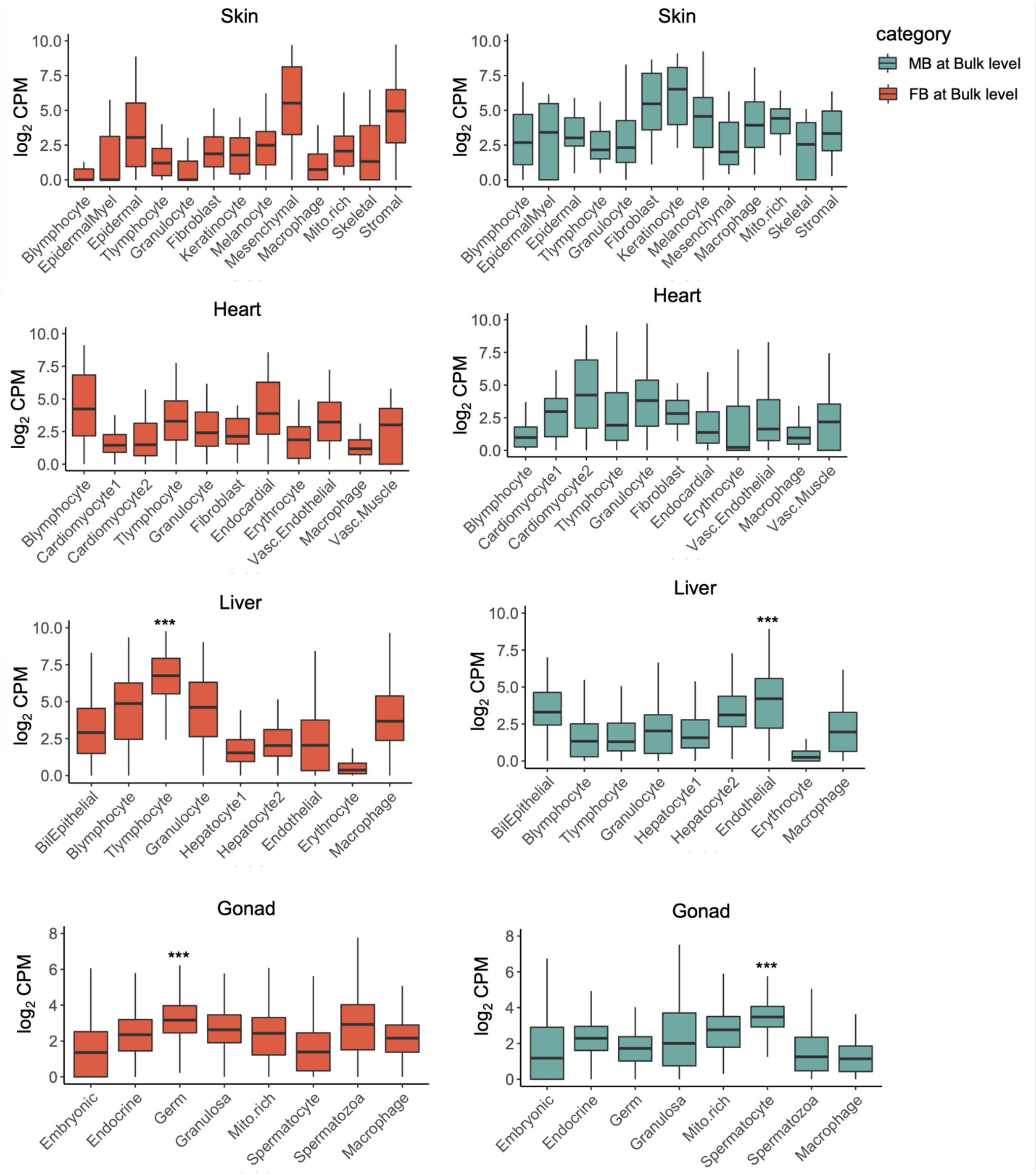

Fig. S8. The ratio of nonsynonymous to synonymous substitutions (d_N/d_S) for all gonad sex-biased genes at the bulk level subdivided based on their degree of expression fold change (highly female-biased genes (FC ≤ −5), moderately female-biased genes (−5 < FC ≤ −3), lowly female-biased genes (−3 < FC ≤ −1), lowly male-biased genes (1 ≤ FC < 3), moderately male-biased genes (3 ≤ FC < 5), and highly male-biased genes (FC ≥ 5). Significance values are calculated using paired Wilcoxon's signed-rank tests (*** $p < 0.001$, ** $p < 0.01$, * $p < 0.05$).

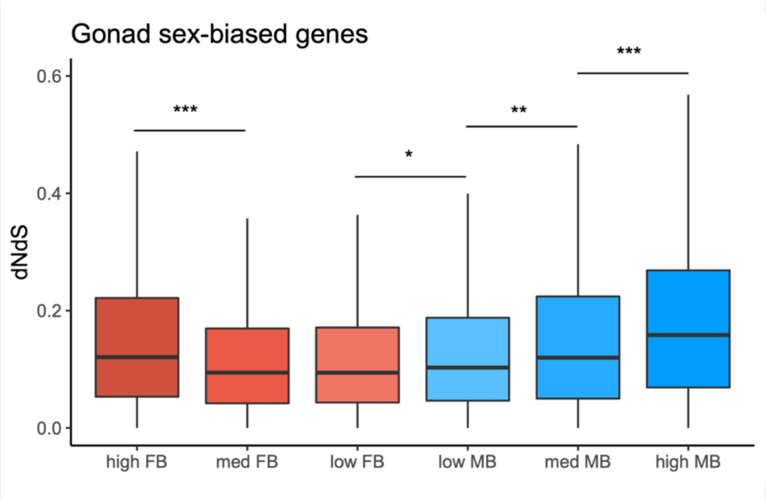

Table S1. Number of cells recovered for each sample.

Tissue	Female1	Female2	Female3	Male1	Male2	Male3
Skin	3193	1491	3747	917	503	2255
Heart	6735	8966	6150	4615	1163	1294
Liver	5814	5991	6075	4985	6070	6835
Gonad	3162	2063	1492	7973	6541	6783

Table S3. Differential cell type abundance between males and females. Shown are female and male proportions for each identified cell type within each tissue and the log_2 fold changes (female:male) in proportions. Significance based on two-proportions z-tests.

Tissue	Cell Type	Female prop.	Male prop.	log_2FC (F:M)	*p* value
**Skin**	Mesenchymal stromal	0.082	0.033	1.286	0.001
	T lymphocyte	0.215	0.143	0.587	0.001
	Keratinocyte	0.076	0.051	0.584	0.001
	Epidermal (myelin-rich)	0.016	0.012	0.365	0.161
	Mitochondrial-rich	0.088	0.071	0.307	0.002
	Epidermal	0.070	0.070	-0.014	0.920
	Skeletal muscle	0.013	0.013	-0.018	1
	Granulocyte	0.021	0.022	-0.064	0.790
	Stromal	0.107	0.128	-0.254	0.001
	Macrophage	0.040	0.049	-0.302	0.023
	Fibroblast	0.238	0.303	-0.346	0.001
	B lymphocyte	0.017	0.025	-0.547	0.005
	Melanocyte	0.017	0.079	-2.178	0.001
**Heart**	B lymphocyte	0.060	0.027	1.120	0.001
	Fibroblast	0.243	0.179	0.446	0.001
	T lymphocyte	0.124	0.099	0.321	0.000
	Cardiomyocyte 1	0.162	0.140	0.211	0.000
	Macrophage	0.144	0.136	0.076	0.129
	Vascular smooth muscle	0.010	0.010	-0.015	0.994
	Granulocyte	0.009	0.011	-0.277	0.163
	Erythrocyte	0.179	0.245	-0.451	0.001
	Endocardial	0.033	0.062	-0.915	0.001
	Vascular endothelial	0.020	0.039	-0.947	0.001
	Cardiomyocyte 2	0.016	0.051	-1.676	0.001
**Liver**	Erythrocyte	0.043	0.024	0.830	0.001
	T lymphocyte	0.157	0.094	0.742	0.001
	Hepatocyte 1	0.496	0.464	0.096	0.000
	Granulocyte	0.017	0.019	-0.163	0.164
	Biliary epithelial	0.027	0.033	-0.270	0.002
	B lymphocyte	0.038	0.046	-0.277	0.000
	Macrophage	0.034	0.046	-0.435	0.000
	Hepatocyte 2	0.182	0.261	-0.518	0.001
	Endothelial	0.005	0.012	-1.254	0.001
**Gonad**	Germ	0.196	0.000	30.871	0.001
	Mitochondrial-rich	0.111	0.001	7.118	0.001
	Macrophage	0.210	0.003	6.017	0.001
	Granulosa	0.219	0.005	5.352	0.001
	Embryonic Yolk Sac	0.040	0.004	3.271	0.001
	Endocrine	0.207	0.022	3.250	0.001
	Spermatocyte	0.017	0.836	-5.660	0.001
	Spermatozoa	0.001	0.129	-7.760	0.001
